# The value of “us”—Expressions of togetherness in couples where one spouse has dementia

**DOI:** 10.1111/opn.12299

**Published:** 2019-12-30

**Authors:** Anna Swall, Christine Williams, Lena Marmstål Hammar

**Affiliations:** ^1^ School of Education, Health and Society Dalarna University Falun Sweden; ^2^ College of Nursing Florida Atlantic University Boca Raton FL USA; ^3^ Division of Nursing Department of Neurobiology, Care Sciences Karolinska Institute Stockholm Sweden; ^4^ Care and Social Welfare School of Health Mälardalen University Västerås Sweden

**Keywords:** caring, communication, couples, dementia, nursing

## Abstract

**Background:**

Living with dementia involves both illness and health, and self‐care and care from others. As most persons with dementia live in their own homes, dementia affects not only the person with the disease, but also family, commonly the partner. Research shows that spousal carers feel as though they are losing their partners since they can no longer share thoughts, feelings and experiences as a couple.

**Aim:**

The aim of the study was to describe the sense of togetherness of the spouses when one spouse has dementia.

**Method:**

The sample consists of 18 recorded conversations between 15 persons with dementia and their spouses. The filmed conversations were transcribed verbatim and then analysed using qualitative content analysis.

**Findings:**

One overarching theme arose: *Dementia preserved and challenged the value of* “*us*.” It can be challenging for a couple in which one partner has dementia to preserve a sense of togetherness and to have the relationship they wish for.

**Conclusion:**

Based on our results, we suggest that practitioners should help couples to strengthen their bond as a couple so as to maintain a sense of well‐being. Future studies should examine couplehood under differing conditions, such as long‐ versus short‐term relationships. Prior relationship quality may also be a factor that influences the sense of couplehood following a serious health challenge, such as dementia.

**Implication for practice:**

When spouses were able to live together, their relationship was enriched at many levels. Their love for each other strengthened them as a unit – as an “us” – where togetherness seemed to be strong. Future studies need to examine whether the sense of couplehood varies depending on the length of the relationship (i.e., a relationship of many years or a relatively new relationship).

**What does this research add to existing knowledge in gerontology?**

- The study shows how couples where one partner has dementia may be strengthened by their history together as a couple.
- The study shows that the couples' conversations pointed to the fact that their relationship was full of rewarding experiences that they managed to have together despite the dementia.
- The study shows that couples who lived together felt close and that life had meaning because they were experiencing the challenge of dementia together.

**What are the implications of this new knowledge for nursing care with older people?**

- Nurse can encourage spouses who live together to share memories and experiences which could benefit their relationship despite dementia.
- For a more person‐centred perspective, nurses could advantageously include the partner when planning care.
- At the organisational level, couples‐focused care can be implemented.

**How could the findings be used to influence policy or practice or research or education?**

- As the majority of research focuses on the negative impacts of dementia on couples and on impairments because of dementia, these findings contribute to understanding the possibility of rewarding relationships despite dementia.
- Future research needs to examine whether the sense of couplehood varies depending on the length of the relationship (i.e., a relationship of many years or a relatively new relationship).
- Nurses can be taught to be aware that prior relationship quality may also be a factor influencing the sense of couplehood that follows a serious health challenge such as dementia.
- Nurses who care for couples living with dementia need to learn that spouses can experience love and togetherness that may influence the care relation.

## 1 BACKGROUND

According to the World Health Organization (WHO), nearly 10 million people will develop dementia every year worldwide, and the current number of 50 million people diagnosed with dementia is expected to triple to 152 million people in the next 30 years (WHO, 2017). Dementia is associated with an extensive range of disabilities such as memory loss, communication problems, disorientation and behavioural symptoms such as resistance and aggression. As a result, it is the leading cause of dependency in everyday living with needs increasing as the disease progresses (Alzheimer's Association, 2018; Alzheimer's Disease International, 2013). Eventually, this leads to the need for 24‐hr supervision (Cerejeira, Lagarto, & Mukaetova‐Ladinska, 2012; Haibo et al., 2013; Rockwood et al., 2015). These extensive care needs burden the healthcare system and subsequently the economy (Allergi et al., 2007; Hugo & Ganguli, 2014; Schaller, Mauskopf, Kriza, Wahlster, & Kolominsky‐Rabas, 2015).

Living with dementia involves both illness and health, and self‐care and care from others. At times, people with dementia act independently, but at other times, they may not have the ability to recognise their shortcomings, for example managing their personal hygiene (Emery Trindade, Santos, Lacerda, Johannessen, & Nascimento Dourado, 2018; Kitwood, 1997). Research shows that living with dementia induces feelings of powerlessness over one's life, uncertainty in an unfamiliar world (Emery Trindade et al., 2018) and a desire to be included, supported, appreciated and respected (Tranvåg, Petersen, & Nåden, 2013). Hedman, Hellström, Ternestedt, Hansebo, and Norberg (2014) stated that to preserve the person's sense of self is to preserve a part of his/her identity. McCormack and McCance (2017) describe personhood as the most important component when practicing person‐centred care. Personhood includes a sense of self as a human, with feelings and emotions, desires and thoughts. In addition, to exist in relations to others, to be a social being, to be recognised and respected. Edvardsson, Winblad, and Sandman (2008) describe person‐centred care as when the lived experience of the person with dementia, including the person's uniqueness and worth, is included in the caring process.

As most persons with dementia live at home (Ludecke et al., 2018; World Alzheimer Report, 2015), dementia affects not only the person with the disease, but also the family, commonly the partner (Johannessen, Helvik, Engedal, & Thorsen, 2017; Liu et al., 2017; Wadham, Simpson, Rust, & Murray, 2016). Research shows that spousal carers feel they are losing their partners due to an inability to share thoughts, feelings and experiences as a couple (Ask et al., 2014; Pozzebon, Douglas, & Ames, 2016). Further, they find themselves in stressful situations and perceive themselves to have a lower quality of life than the person with dementia (Ask et al., 2014; Wadham et al., 2016). The marriage is often strained, and carers feel trapped and lonely in the relationship, with little time for themselves (La Fontaine & Oyebode, 2014). However, Hellstrom, Nolan and Lundh (2005) and Merrick, Camic, and O'Shaughnessy (2016) found that both the spousal carer and the person with dementia are motivated to maintain their “couplehood” (Hellstrom et al., 2005; Merrick et al., 2016). The concept “couplehood” was first described by Hellstrom et al. (2005) and was formulated from the definition of personhood first described by Kitwood (1997). The meaning of couplehood suggests that couples affected by dementia should be viewed as a unit rather than two separate individuals. Further, strategies to preserve and maintain their relationship should be developed based on their sense of couplehood: for example, promoting being and doing things together (Hellstrom, Nolan, & Lundh, 2007). Bielsten and Hellstrom (2017a, 2017b) and Merrick et al. (2016) suggested strategies for maintaining couplehood to include engagement in the relationship and co‐activities. Daley, O'Connor, Shirk, and Beard (2017) wrote that carers who viewed their relation in terms of “we/us” instead of “I/me” experienced their situation as being less demanding, they were more satisfied in their relationship, and they perceived more mutual compassion in the relationship. Hernandez, Spencer, Ingersoll‐Dayton, Faber, and Ewert (2017), as well as Hellstrom et al. (2005), suggested the importance of doing things both together and separately so that both the spouses and the persons with dementia can preserve a sense of self. Also, preserved couplehood makes it easier to work through problems together, to move on and to let go (Bielsten & Hellstrom, 2017b; Daley et al., 2017; Hellstrom et al., 2007). Wadham et al. (2016) described the importance of decade‐long relationships as being a basis for sustaining couplehood during challenging times. Williams and colleagues (Williams, 2015; Williams, Newman, & Hammar, 2017) stated the importance of couples being able to engage in emotional communication in order to sustain their relationship. They developed a home‐based intervention—Caring About Relationships and Emotions (CARE)—to improve communication and to support the relationship. In a study by Williams et al. (2017), communication improved for both the spousal caregiver and the partner with dementia after they took part in the 10‐week CARE intervention.

Life together as a couple when one spouse has dementia is described in the literature as being primarily one of frustration, isolation and separation. There is a gap in the literature regarding couples' sense of togetherness, and their living as a unit rather than two separate individuals, and being and doing things together (Balfour, 2014; La Fontaine & Oyebode, 2014).

The aim of the study was to describe the sense of togetherness of the spouses when one spouse has dementia.

## 2 METHOD

### 2.1 Design

This was a secondary data analysis of existing data from a study of the effects of a 10‐week, dyadic, home‐based communication intervention on verbal and non‐verbal outcomes in couples in which one individual was affected by Alzheimer's disease or a related dementia. The researcher instructed 15 spousal carers to be open and to avoid correcting, condescending or arguing with their spouses. Further, CGs were taught to use clear, succinct and respectful communication, and to avoid testing their partner's memory. The persons with dementia had the opportunity to practice conversation with a member of the research team who was trained in communication deficits associated with dementia as well as the intervention. After the pre‐ and post‐test visits and after each of the 10 weekly intervention sessions, couples were asked to engage in a 10‐min conversation on a topic of their choice. Conversations were recorded by the researcher. After setting up a video recorder on a tripod, the researcher left the room. After 10 min, the researcher returned and turned off the recorder. At a later time, recorded conversations were transcribed. For this study, a qualitative analysis of transcribed conversations was conducted to increase understanding of the spousal carers' sense of togetherness based on their verbal interactions with their partners with dementia.

### 2.2 Sample and settings

Couples were recruited for the parent study from attendees at a memory centre day programme in south Florida in the United States. The sample for this study consisted of eighteen recorded conversations selected from conversations based on the substance of verbal communication between the person with dementia and the spouse. For example, conversations where the person with dementia was too tired or did not interact were not included in the analysis. Participants were White. The average age for carers was 77 and 80 for spouses. Most couples had long‐term marriages with an average of 47 years, were middle‐income and generally well‐educated. Most of the carers (68.8%) reported that they performed the role of caregiver for an average of 4 years. Severity of dementia for affected spouses was moderate (*M* = 16.8, standard deviation = 7.4) as measured by the Mini‐Mental State Exam (Folstein, Folstein, & McHugh, 1975).

### 2.3 Analysis

The filmed interactions between the person with dementia and spousal carer were transcribed verbatim including verbal (speech) and non‐verbal communication (body language, facial expressions) and then analysed using qualitative content analysis as described by Graneheim and Lundman (2004). To get a true sense of the entire text, each transcript was read as a whole, and then, the text was categorised into meaning units that included sentences and phrases related to the aim of the study. The meaning units were then condensed and abstracted into codes (Table 1). Events, expressions and phenomena (verbal and non‐verbal) that had connection to the aim of the study became visible in codes that emerged from the text. The steps in the analysis provided opportunities to move back and forth between the different levels of the analysis, which was helpful in the structuring of the text. The codes were then compared to check for similarities and differences and then further abstracted into two themes, each with two sub‐themes and finally one overarching theme that encompassed the themes (Table 2). The overarching theme, theme and the sub‐themes represented the latent content of the text on an interpretive level.

**Table 1:** Example from the analysis process

Meaning unit	Condensation	Code
Man and woman sitting in a sofa. The man is holding his arm around the woman's shoulder. The woman holds her hand on the man's leg. Man (carer): turns his head to face the woman. Saying: “Yes. That's what they want to find out, is how you and I communicate. You know how we do?” Woman (person with dementia): Looks at the man and smiles and says: “no” Man: (holding the womans cheek and kisses her) “that's how.” Woman: “now we are communicating she says and laughs.” Man: looks into the camera and says: that's right Woman: kisses the man on the cheek, and says: I just communicated. Man: Turns his head and looks at the woman, and says: “actually you communicate very well with Bridget (daughter) and me and people you know, you communicate very well. And we're getting along fine.” Woman: Smiles and says: “Oh baby I love you.” Man: smiles and says: “I know.” Woman: Looks at the man and says: “I don't know what's happening here with what I do but.” She is shaking her head Man: Looks at the woman and says “I know, that's okay.” Woman: “I don't give a damn” she raises her voice and looks at her knee. Man: Takes the woman's hand and looks at her. “Okay, good enough. It doesn't matter. It's not a test that whether you're going to pass it or not pass it. It's just a measurement. To measure what level you're at, that's all.”	The man and woman communicate through kisses. The man thinks the woman communicated well with him and Bridget. They express their love for each other, verbal and non‐verbal. The man affirms the woman when she does not know what is happening by saying “It is ok.”	Unconditional love

**Table 2:** Sub‐themes, themes and overarching theme

Sub‐theme	Theme	Overarching theme
Strengthened in their love for each other when together Altruistic in their support for their spouses Life is a blessing Frustrated existence	Their love for each other supported togetherness Taking the good with the bad	Dementia preserved and challenged the value of “us”

### 2.4 Ethical considerations

The study was approved by the University Institutional Review Board (IRB) at Florida Atlantic University. For the parent study, proxy consent was obtained from the participants' spouses, and assent was sought in all contact with the participants.

## 3 FINDINGS

The findings resulted in one overarching theme *Dementia preserved and challenged the value of* “*us*,” which in turn were developed into two themes and four sub‐themes (Table 2).

### 3.1 Dementia preserved and challenged the value of “us”

When one partner has dementia, it can be challenging for a couple to preserve a sense of togetherness and to have the relationship they wish for. In many situations in this study, they managed to preserve what had been “us” and their feelings as “before.” In other situations, they failed to be “us” and struggled to find the feelings of security that come with “us.” “Us” is the link that keeps the couple together and that keeps them functioning in everyday life.

### 3.2 Their love for each other supported togetherness

The theme *Their love for each other supported togetherness* was built upon the sub‐themes *Strengthened in their love for each other when together* and *Altruistic in their support for their spouses*. It focused on the good things in life, on functioning together despite the memory loss of one spouse and on supporting the person with dementia despite the spouse's own wishes or needs. When the spouses were together and doing valuable things as a couple, they found strength in their relationship.

#### 3.2.1 Strengthened in their love for each other when together

Spousal carers often sought confirmation from their spouses that they—the partners with dementia—felt they were receiving the care they needed and that the love the spousal carer gave was enough to provide the person with dementia with a sense of safety and security.

Man (carer): sitting in a sofa, looking at the woman sitting on a chair next to the sofa. Saying: “Yes. So uh, you're doing fine, just stay happy, stay happy” he says and smiles. Woman: looks at him in a worried manner saying: “I guess the main thing for me is that uh I want you to be happy with me.” Man: takes her hand saying: “I am. I'm always happy with you. That's why I say I'm happy with you whether you answer a question or you don't answer a question.” (Laughter from both looking at each other). “It doesn't matter to me. I don't care if you know what day it is. I know what day it is, and if you need to know you can ask me, and I'll tell you.” Woman: smiles looking at him, saying: “I do that. I do that.” Her smile disappeared, saying: “sometimes too much…”

Man: Looks at her, still holding her hand saying: “Sometimes you do, and it's never too much. If you want to know what day it is, you ask and I'll tell you. And if I don't know the answer, I'll tell you I don't know the answer too.” Woman: smiles a little saying: “That's okay.” (105205)

Spouses talked about their life and their relationship as being full of good things that they managed to do together despite dementia: for example, trips they once made and what has happened in the lives of their children and grandchildren. To have each other was often the link that held them together despite difficulties in everyday life. Their relationship meant that everyday life made sense because they experienced it together. The spousal carer acknowledged the person with dementia in many situations and provided a sense of security—that everything was fine despite memory loss. Further, the spousal carer helped the spouse with dementia to remember when the need arose and when they supported each other. In some contexts, the person with dementia needed to hear that everything was fine despite memory‐related problems.

Man (Carer): sitting around a table looking at the woman sitting towards him saying: “They're basically the same. They want to see if your memory is getting better, is getting worse, or it stays the same.” Woman: Looking at the man in a surprised way, saying: “Yeah. Have I… have I fallen down on you?”

Man: looks at her and says: “No, no you are just about the same as you were. You're fine… as long as you remember me baby.” (smiling big). Woman: smiles big and says: “Yes and not only that. I remember my name too.” (105205)

Some spouses were deeply committed to ensuring that their loved one had the best in life and therefore adapted any situation so that their spouses' needs or wishes could be fulfilled. The spousal carer used endearments such as “sweetheart,” “angle” and “my love” to demonstrate how much the carer appreciated the spouse's love despite changes in functional abilities due to the disease.

#### 3.2.2 Altruistic in their support for the spouse

By questioning and reassuring the person that they would have everything they needed and wished for in the current situation, and by prioritising the loved ones' needs, they strengthened their relationship.

Woman (Carer): Sitting around a table eating together with the man, saying: “I'm going to give you a sweater.” I'll give it to you, then we'll go out. She leaves and comes back with a sweater and lays it over his shoulders and says “Here, my angel. You are the sweetest guy in the world.” as she looks at him and rubs his shoulders. “I have told you this” (looking in his eyes, and he looks back). “And I love you, love you, love you…” (smiles and rubs his shoulders). Returning to her chair and says: “I'm happy that you're hungry.” She gives him food with a spoon saying: “Here, Sweetie.” Looking at him saying: “Do you know what you are eating? Fish, right? That's the reason I got it for you, because you love fish.” (smiles and look at him. He looks at the plate). She looks at his plate saying: “Oh, I'm so sorry! Forgive me. You want some milk… I have milk” (standing up to pick up milk and a napkin), “I have everything for you, my angel. Yes, I'm coming my angel, I'm coming. Here's a napkin for you…” (106206)

### 3.3 Taking the good with the bad

The theme “Taking the good with the bad” encompassed the sub‐themes *Frustrated existence* and *Life is a blessing*. Carers acknowledged that life's blessings and frustrating moments fluctuated just as they do in everyone's lives.

#### 3.3.1 Frustrated existence

The sense of togetherness was challenged when the communication between spouses varied in quality. Sometimes, their conversations resulted in frustration and pressure from the person who did not remember what the healthy spouse wanted to share.

The man and the woman are sitting in each armchair with a small table between them. The man (carer) says looking at the woman: “Who was Moses? Was he our cat?” Woman: points at the floor saying: “Right over there.” Man: Lean his head looking at her, saying: “Mandy, Mandy was Moses our cat?” Woman looks at him nodding and says: “Yeah.” Man puts his hand on his chin and looks at her: “Okay, let's see. Did you ever smoke?” Woman looks at him in a confused way saying: “What?” Man looks at her and repeats: “Did you ever smoke?” Woman looks at him in a offensive way: “Did I ever smoke yet?” Man nods and says: “Did you ever smoke cigarettes?” Woman roll her eyes saying: “Well, not a big one.” Man looks at her and repeats: “No, did you ever smoke cigarettes?” Woman looks at him confused and little irritated saying: “The size of?” Man articulate with his hands saying: “Well, you used to smoke.” Woman looks down at her knee saying: “Yeah.” Man looks at her saying: “You gave it up though.” Woman looking at her knee saying: “Yeah.” Man: “Why did you give it up?” Woman looks at him saying: “When.” Man looks into the camera saying: “Okay.” Woman put her hands together and looks at them saying: “Now you… through that” (talks incoherently). Man looks at her saying: “No.” Woman looks at her hands saying: “Don't ask me anymore.” (307407)

Apparent frustration and guilt were also noted when the loving spouse had to take on extra responsibility and ask friends for help with things that in the end would benefit them both: for example, running errands.

Woman (carer) sitting around a table. Looks at man saying: “because sometimes you have a little difficulty standing up, and she's (friend) going to help with the wheelchair when we have to go out, with the walker, so I don't have to lift it, and she'll do it for me. So, that's what's doing. Other than that…” Man looks at her angrily saying: “horrible…” Woman looks at him saying: “What's so horrible about that?” Man looks down in the table saying: “Everything.” (308408)

Some situations could evoke thoughts that occasionally could describe their common life:

Man (carer) sitting on the end of a sofa. The woman sitting on the other end. He looks at her laying his hand closer to her and says: “What does that mean, what you're saying really is, that our relationship has changed as we've aged or grown old together or put in a happy way, or as we go on life changes, and we've changed with it. Now our relationship to each other has changed.” (110210)

Their daily lives together consisted of enriching elements but also frustrating situations that affected both the person with dementia and the spouse, but which they often managed to accept and live with.

#### 3.3.2 Life is a blessing

This sub‐theme focused on life's blessings and on acknowledging life‐enriching experiences when the situation was frustrating for both spouses and challenged their togetherness. To be able to function together as a couple in everyday life was seen as a blessing even when one spouse had dementia. Couples were able to value the good things in life, such as support from friends and relatives, and the care the couples received from those around them. Being able to live in the moment with each other supported these perceptions.

Woman (carer). The couple is sitting on either side of a table. The woman looks at spouse says: “So from all of your things, from all of your activities, what do you enjoy the most?” Man looks at her smiling and says: “Being with you.” Woman laughs and says: “I love you.” Man: (blows kiss)

Woman: blows kiss, smiles and looks at him saying: “I enjoy being with you. We're almost together 24/7, but it's still fun, I look forward to that.” Reaches her hands towards him over the table saying: “I remember before we retired, you used to think about when you retired just not having to work and being together.” Man reaches out his hands in the air, looks at her, smiles and says: “And here we are.”

Woman Looks at him saying: “Here we are spending good times together.” Man nodding and says: “yes, yes.” Woman looks at him saying: “Trying to live in the moment.” Man looks at the table nodding and says: “Right, right.” Woman looks at him saying: “Not in the past, not in the future, but only in the moment.” (305405)

Spouses' conversations about life's blessings could also include a mixture of blessings and challenges, but often there was an accepting attitude towards life in general. Some spouses found their communication with each other to be the most important aspect of their relationship.

## 4 DISCUSSION

The results showed that couples where one person has dementia found strength in each other and in their commitment to their relationship. Their conversations with each other pointed to the fact that their relationship was full of rewarding experiences that they managed to have together despite the dementia. Memories were about family and friends as well as trips that they had taken. The fact they lived together brought them close, despite the challenges that then came with everyday life. At the same time, life had meaning because of the fact they were experiencing it together. According to Wadham et al. (2016), it was important for couples to maintain a shared identity of “us and we” so that they could continue to feel a sense of togetherness—of being “one”—when one spouse was affected by dementia. The present study shows a similar description of spouses' lives together with the value of “us” strengthened as a result of everyday experiences with the ups and downs related to dementia. Hernandez et al. (2017) also found that both parties in the relationship experienced a sense of unified identity despite the effects of dementia‐related memory loss. These findings are consistent with Hellstrom et al.'s (2005) description of couplehood. Merrick et al. (2016) further stated the importance of upholding couplehood. Bielsten and Hellstrom (2017a, 2017b), together with Han and Radel (2016) and Riley, Evans, and Oyebode (2018), emphasised the importance of strategies based on dedication to the relation as well as to activities experienced together. This was also evident in our results when the couples talked about memories. The sense of couplehood—the “we/us” notion as Daley et al. (2017) describe it—resulted in the spousal carer finding the care for his/her partner to be less demanding (Daley et al., 2017). The results demonstrate the challenges that came with holding a conversation and other such communication problems as the dementia progressed. Previous studies confirm this, and Evans and Lee (2014) stated that changes in relationship roles and an inability to maintain togetherness, reciprocity and intimacy challenged the relationship. Further, Murray, Schneider, Banerjee, and Mann (1999) described a loss of companionship that arose because of communication problems.

Hernandez et al. (2017), as well as Merrick et al. (2016), further state the importance of a decade‐long relationship to create bonds that would sustain couplehood during challenging times, such as came with dementia. This raises questions regarding couples who do not have a long relationship and regarding how dementia affects their sense of commitment and togetherness. In our sample, couples were married 47 years on average with a range of 17–65 years; as such, there were no short‐term relationships. In the future, the phenomenon of couplehood following a dementia diagnosis in a short‐term relationship warrants further study. We suggest, based on our results and in line with Hernandez et al. (2017), that practitioners should help couples to strengthen their bond so as to maintain a sense of well‐being and to carefully assess the well‐being of couples whose relationship is relatively new at the time one partner is diagnosed with dementia. A way of doing this might be to use the CARE intervention on emotional communication as described by Williams et al. (2017). Further, Lasrado et al. (2018) suggested a home‐based couple management guide (DemPower) that focused on activities that can be done together as a tool to increasing the sense of togetherness. It is possible that couplehood and togetherness in situations where one partner has dementia may strengthen the sense of person‐centredness for both the person with dementia and the spouse. Couples have unique life experiences together that are meaningful in their daily lives. Person‐centredness is described by McCormack & McCance (2006) as living one's own life while doing meaningful activities despite symptoms of dementia. Further, Edvardsson et al. (2008) described person‐centredness in the caring context, person‐centred care, which is commonly seen as a standard for high‐quality care, especially in the care of persons with dementia (Manthorpe & Samsi, 2016; Ruggiano & Edvardsson, 2013). It is possible for persons with dementia to be cared for by their spouses in a unique person‐centred way because of their life experience together in a manner that only they could achieve. It is also possible that persons with dementia in some couples can be helped in a person‐centred way so that they can live with their spouses as long as possible because of their deep understanding of each other and each other's lives. This understanding and knowledge of each other are unique for the couples and hard for others to know. In that way, person‐centredness between couples who live together may be deeper than ever and could mean that in good relationships the care from a spouse is optimally person‐centred.

## 5 LIMITATIONS

To record the conversations spouses had about their life together, we determined video recording to be a suitable method. Recordings of unstructured conversations about their daily lives without being directed by the researcher in terms of topics led, on occasion, to deep conversations between spouses about their love for each other and other intimate matters in their lives. The fact is that there is a risk people change their behaviour when they are aware they are being observed. However, Latvala, Vuokila‐Oikkonen, and Janhonen (2000) state that the participants usually acclimate to the presence of the video camera and start to behave as though they are not being filmed. Our perceptions were that the spouses often had trouble finding a subject to talk about in the beginning, but once the conversation began to flow, they did not seem to notice the camera. Further since the conversations between the spouses took place with no researcher present, participants were free to choose their topics and were possibly free to open up and talk about meaningful things with their spouse.

The collection of data by way of video observations provided opportunity to repeat the sequence of analysis and to increase trustworthiness. Qualitative content analysis (Graneheim & Lundman, 2004) provided opportunity to move back and forth between the whole text and parts of the text at different levels of abstraction as well as to move from the transcriptions and the video recordings so as to gain trustworthy video observations that would confirm the analysis. In addition, all authors participated in the process of analysis so as to increase trustworthiness (AS, LMH, CW). This study focused on togetherness and thus did not include conversations on other topics. Other perspectives may have been found if the researchers focused on different interactions. Persons with dementia commonly have problems in expressing and interpreting communication, and problems of misunderstanding and irritation could appear from both partners. Between couples, both facilitative communication, such as expressions of love and affection, and disabling or disaffirming communication are commonly included in interactions. In our study, expressions of togetherness were clear in the video observations even though disabling communication could appear as well. In this study, we focused on what might enable their interaction and their lives together, and expressions of togetherness was one part that seemed valuable.

## 6 CONCLUSION AND IMPLICATION FOR FUTURE RESEARCH

When spouses were able to live together, their relationship was enriched at many levels. Their love for each other strengthened them as a unit—as an “us”—where togetherness seemed to be strong. There were also times when each spouse might suffer in a different way depending on the situation. Future studies need to examine whether the sense of couplehood varies depending on the length of the relationship (i.e., a relationship of many years or a relatively new relationship). Prior relationship quality may also be a factor influencing the sense of couplehood that follows a serious health challenge, such as dementia. Variations in environment could also affect the relationship between couples after the onset of dementia. The degree of family and community support, economic resources and carers' prior health may influence their ability to create a sense of “we/us.” A future analysis of the recorded conversations could focus on the disabling communication, which is interesting and important as well.

7

**Implication for practice**

- When spouses were able to live together, their relationship was enriched at many levels.
- Their love for each other strengthened them as a unit – as an “us” – where togetherness seemed to be strong.
- Future studies need to examine whether the sense of couplehood varies depending on the length of the relationship (i.e., a relationship of many years or a relatively new relationship).

## CONFLICT OF INTEREST

The authors declare no conflict of interest.

## AUTHOR CONTRIBUTIONS

Study design: CW; Data analysis: AS, CW, LMH; Manuscript preparation: AS, CW, LMH.
